# On the Sarcocele of Egypt, from Mons. Larrey's Relation Chirurgicale de l'Armé de l'Orient
*We are indebted to a valuable correspondent for this translation, from the only copy of M. Larrey's work yet in England, of an interesting history of a most extraordinary disease. Under the term *sarcocele* a disease is here described differing essentially from the affection, which in this country has been designated by that appellation. The sarcocele of Europe is a disease of the body of the testicle, liable to become seriously active from external accident, from irritating applications, and from incisions made into it when mistaken for hydrocele. But we are assured in this memoir, that the sarcocele of Egypt and other hot climates, is a carneous mass enormous in size, possessing little sensibility, having no other connection with the testicle than being in its neighbourhood; bearing the potential and even the actual cautery, and suffering setons to be passed through it, with impunity : and occurring also in the female. The sarcocele of Europe is an organic disease of the testes; (Vid. Jour. for March, p. 276) the sarcocele of Egypt is a disease of the scrotum and capsulæ of the testes in the male, and of the common teguments and cellular substance of the labia pudendi of the female, connected with elephantiasis, and being, possibly, one form of that disease. Ed.


**Published:** 1811-04

**Authors:** 


### Medical and Physical Journal.

VOL. XXV.] APRIL, 1811. [NO. 146.

Printed for R. PHILLIPS, by E. Hemsted, Great New Street, Fetter Lane, London.

*For the Medical and Physical Journal.*

### *On the Sarcocele of Egypt, from* MONS. LARREY'S *Relation Chirurgicale de l'Armé de l'Orient.**

THE word Sarcocele is derived from the Greek (σαρξ κηλη), Fabricius ab Aquapendente, Fabricius Hildanus, Lanfranc, Fallopius, André de Lacroix, and others, have described this disease under the name of caro adnata ad testem. Since these authors, whose observations seem to relate to the sarcocele of warm climates, modern surgeons have confounded it with diseases of the testis, such as swelling, inflammation, scirrhus, hydrocele and hydrosarcocele. The etymology of the word sarcocele, and the signification which antient authors seem to attach to it, proves that this denomination

* We are indebted to a valuable correspondent for this translation, from the only copy of M. Larrey's work yet in England, of an interesting history of a most extraordinary disease. Under the term *sarcocele* a disease is here described differing essentially from the affection, which in this country has been designated by that appellation. The sarcocele of Europe is a disease of the body of the testicle, liable to become seriously active from external accident, from irritating applications, and from incisions made into it when mistaken for hydrocele. But we are assured in this memoir, that the sarcocele of Egypt and other hot climates, is a carneous mass enormous in size, possessing little sensibility, having no other connection with the testicle than being in its neighbourhood; bearing the potential and even the actual cautery, and suffering setons to be passed through it, with impunity: and occurring also in the female. The sarcocele of Europe is an organic disease of the testes; (Vid. Jour. for March, p. 276) the sarcocele of Egypt is a disease of the scrotum and capsulæ of the testes in the male, and of the common teguments and cellular substance of the labia pudendi of the female, connected with elephantiasis, and being, possibly, one form of that disease. ED.

 belongs belongs exclusively to that disease which distends beyond measure the teguments external to the testicle, particularly the scrotum and dartos; and gives to these parts an extraordinary volume or size. The great number of individuals I have seen attacked with this complaint in Egypt confirms me in this opinion, and induces me to trace the causes, symptoms, progress, and effects, and to point out the means of relief, which are in the power of art.

My researches into the nature of sarcocele induce me to believe that it is confined to warm climates, at least, that it is very rarely met with in cold regions, for the great number of examples which we meet with in Europe are the produce of Asia or Africa. The scrotal tumour of the minister, Charles Delacroix, is perhaps a single instance of sarcocele well marked which has taken place in our climates, and the volume of this tumour was small, in comparison of the cases of sarcocele related in the German Ephemeris for 1692, in Dionis's Chirurgical works, the Bibliotheque de Medicine, tome IX, and of those which I have seen with astonishment in Egypt, some of which weighed at least fifty pounds. I shall relate some of those cases which are most worthy of remark. By sarcocele, properly so called, I mean that tumour which is formed in the scrotum, and is a fleshy mass, broad at its inferior extremity, and attached to the pubis by a peduncle or neck more or less extensive.

Externally it is rugous and irregular, separated by furrows or cavities, corresponding with the mucous cryptæ, or the roots of the hairs; yellow scaly incrustations are usually found on its surface, more particularly if the sarcocele be of long duration. When these crusts fall off, there are underneath several small ulcers, which discharge an ichorous fluid. The tumour is indolent and harder at some parts than at others; it may be felt and pressed upon in any direction without exciting the smallest pain.

The patient is no otherwise incommoded by it than by its weight, and the impediment it proves to walking, which compels him to have it suspended. The urine flows over the tumour without excoriating it.

In the great number of sarcoceles which I have seen, I have remarked that the testicles and spermatic chord were in a sound and natural state, and situated on the sides of the tumour; but the vessels of the testis were usually augmented in size (varicose). If the testicle should participate in the disease, it will be accompanied by the symptoms proper to such affection. It does not, however, seem to me, that the testis is capable of so great an augmentation of size with whatever disease disease it may be affected; for the patient would sink if the morbid alteration were in the testicle, before the sarcocele has arrived even to its second stage.

The alteration of the testicle, in such cases, is the original disease, and to be regarded as distinct from sarcocele, and treated according to its particular character. It is not my design to describe the affections proper to the testis, I am only to relate what I have observed of the sarcocele of Egypt. Labourers, but particularly those whose occupation requires their sitting, as weavers, embroiderers, taylors, are most subject to it; many circumstances seem to contribute to produce the disease. Among the internal causes may be enumerated, depraved humours, inveterate syphylis, which has for one of its symptoms in this country, pustules and pruritus of the scrotum, which is, however, much disregarded by the Egyptians. This singular virus, produced also perhaps by vicious humours, is, probably, the cause of another disease not less distressing, elephantiasis. I have remarked that all those who are affected with sarcocele have symptoms, more or less apparent, of elephantiasis. The subject of a case at the end of this memoir is a striking example. All these causes produce their effects on the cellular membrane and skin of the scrotum, as being most disposed to the attacks of psoric and similar complaints; its laxity, its innumerable mucous cryptæ, and the little sensibility it is endued with, predispose it to tumefaction; the vessels first are surcharged, become weakened in their tone, the scrotum enlarges, and at the same time acquires a density like that of the placenta. The testicle preserves its form and health, but soon ceases to be distinguished, except at the posterior part of the tumour, which continues to increase in every direction, but particularly downwards. The cellular membrane thickens as well as the envellopes of the testicle; and the skin also augments in density and size. The integument which covers the pubis, the inguina, the penis, and the nates, contributes gradually to form the enormous tumour which is produced; thus the skin covered with hair, which protects the pubis, descends considerably below that region. The extremity of the prepuce presents itself in the form of a sort of navel on the anterior surface of the tumour. The urine flows down from the aperture without being projected. The external surface of this fleshy mass becomes rugous and scaly; it retains but little warmth, and the superficial veins are considerably enlarged. Sarcocele may increase to almost any size; a case related in the German Ephemeris weighed more than two hundred weight. The case of a Fellah (agricultor) of Upper

 Egypt,

Egypt, which I shall hereafter relate, was considered to weigh one hundred pounds. I have seen in different parts of Egypt ten or twelve different cases. If these tumours are dissected they seem composed of a dense (couenneuse) substance, with little vascularity and harder in some parts than in others; these have but little sensibility, and when cut do not give much pain. This I was able to remark on extirpating a commencing sarcocele in a cook of the Capuchin convent, at Cairo. At the School of Medicine at Paris, there is a model of a sarcocele, which was not extirpated, but the dissection after death confirms this description. The testicles were sound, and the tumour was formed by their membranes, then preternaturally distended.

An old man, at Cairo, consulted me for an enormous sarcocele which he had had for twenty years, the size of which obliged him to keep his bed. Anxiety to be relieved had impelled him to consult the physicians of his country, who had exhausted their efforts in vain. Cautery, caustics, incisions, and discutients, had been employed. The last person he consulted pierced the tumour through its centre with a large needle and ligature; he suffered but little pain, which proved that the testicles did not participate in this disease. This seton, which was moved daily, produced an abundant flow of serous fœtid matter; (he was also affected with elephantiasis.) The continued discharge of the seton produced but little diminution of the tumour; and as no more was to be expected from it than other means which had been employed, I proposed the extirpation; and when I was about to perform it an order to go to Alexandria, which the English had threatened to attack, compelled me to leave this unfortunate old man to his fate. To the causes I have adduced may be added, bad diet, intemperance, too frequent venery, the immoderate use of warm baths, to which all classes of Egyptians are addicted; living in damp and marshy places, the effects of climate, mode of dress, or injuries of the scrotum, may also contribute to the formation of the disease.

Sarcocele has been hitherto considered to belong exclusively to man; the term is limited to the disease in the genital organs. But we may consider the fleshy tumours which take place in other parts, particularly in the face, where the skin is liable, as well as the scrotum, to be affected by venereal and other diseases, as so many sarcomatous tumours of the he same nature and depending on the same causes. The examples of such tumours are sufficiently numerous. There are also local causes which determine their formation in one part rather than others, such as falls, mechanical injuries of the skin, or the application of chemical corrosives.

No

No author, that I know of, has described a similar disease in the female genitals, although the skin which envellopes these parts, differs but little from the skin of the genital organs of man. The periodical discharge and other resources given by nature to the female, operate, without doubt, against the formation of these excrescences. But a singular case of a woman, named Hammet Fatomi, of Cairo, furnishes me with an example of well marked sarcocele of the labia; I shall relate this case. Every author who has written on sarcocele has described it as incurable, from the ill success they have had by internal or topical remedies. All those who have proposed extirpation have been fearful, or at least have not practised it. M. Imbert Delonnes has the merit of being the first to perform this operation, in boldly extirpating the sarcocele of Delacroix*; I did not know of the success of his operation when I performed a similar one, (the case I have cited) and proposed to extirpate several other enormous sarcoceles, when the army was removed.

When the complaint is recent it may be treated with the remedies hereafter described; but in an advanced stage there is no other resource but extirpation, preceded, however, by remedies proper to remove the cause of the disease. Among the internal remedies antimonials combined with mercurial medicines, and convenient doses of sudorifics, continued for some time, or alternated with small doses of the mineral acids, diluted in some mucilaginous fluid, produce the best effects, but particularly the sulphuric acid, lowered and applied in form of lotion to the parts, or a weak solution of muriate of mercury, or oxide of copper, or muriate of ammonia, the effects of which are increased by gentle and uniform pressure on the disease. The success of these means will be evident in the diminution of the bulk of the tumour, the retraction of the skin, and the amendment of the patient's countenance. If this be the case, the remedies should be continued until the disappearance of the disease. Incisions or caustics seem to me to be useless. I rest my opinion on the little success which the Spanish and English surgeons had in one of the cases related. It is even possible that these means, if followed by the astringent remedies I have mentioned, may produce cancerous ulceration. But after the use of these remedies differently combined, and for a sufficient time, if the sarcocele continues in the same state, I do not hesitate to pronounce the necessity of amputation, and the possibility of performing it without danger. Its necessity is marked by the

---

* See his Memoir.

failure failure of all other means, and the certainty that the disease will continue to increase, and though the inconveniences are not intense, they lead with certainty to the grave.

It now only remains for me to describe how the operation is to be performed. The vessels which supply the tumour arise from the external pudic artery, and some branches of the internal pudic artery. The spermatic arteries are sent wholly to the testes, and are therefore out of the way; and the hæmorrhage which the other vessels produce is not of much importance, and the arteries are easily secured by a ligature when they are divided: The operation is long and tedious, but not highly painful. The removal of the tumour being complete, if the disease even has been complicated with elephantiasis, which I have usually seen, there is no fear that the sarcocele will be reproduced; but the remedies for elephantiasis should be continued.

There are some general precepts for this operation: the testes should be carefully avoided, and also the corpora cavernosa and spermatic cords. Two oblique incisions beginning from the prepuce, one passing down below the testis on each side of the tumour. The parts between the testicles and corpora cavernosa, must be deeply cut with a double edged knife, carefully avoiding the testes, and the portion below the line formed by these incisions should be removed. If there still remain some sarcomatous substance round the penis or testes, it should be dissected away. The corpora cavernosa and testes are to be covered by the skin which is left, and the edges may usually be approximated and confined by ligature, or by plaister and bandage. The parts discharge, retire and cicatrize without difficulty. If hæmorrhage occurs, the vessels should be secured immediately with ligature, or (if their orifices cannot be discovered) by actual cautery. The success of the operation will be improved by continuing the use of internal remedies.

### CASE I.

Jacques Moline, a Copt, and cook to the convent of Capuchins, at Cairo, consulted me for a considerable tumour in the scrotum, which he had had for many years; it was of a pyramidal form, and weighed about six pounds. The right testicle corresponded with the superior part of the tumour and was sound; the penis had almost disappeared; the left testis was confounded with the fleshy mass which formed the sarcocele, and could not be felt: I still doubted if it formed part of the tumour, for he had never felt pain. This swelling was formed of a dense (couenneuse), and in some some parts almost cartilaginous substance. In the middle of the irregular mass the testicle was discovered diminished in size; the wound was properly dressed. The treatment was not disturbed by any untoward accident, and on my departure for Alexandria, I left the patient advancing to his recovery.

### CASE II.

Mahammet Ibrahim, about sixty years of age, was blind and affected with elephantiasis, which he had had for many years. His legs were half as large again as his thighs, and his feet were monstrous. The skin towards the superior part of the leg was smooth and marbled, and there were many varicose veins running on it. The other part was round, with thick yellow rugous incrustations, disposed like scales, separated from each other, particularly at the articulations, by deep ulcerated furrows, which discharged an ichorous fœtid fluid. The crusts were more considerable at the ancles and the heel than other parts. Deep cavities were observable between the toes, and on the sole of the foot. When pressure was made on the parts of the limb which were most swelled, there was no pain produced, nor was any impression left by the finger; the skin and cellular membrane offered all the resistance of cartilage.

This man had lost his sight by the endemic ophthalmia; he was of dark complexion, of weak constitution, and languished out a miserable life. The tumour weighed about seventy-five pounds, it was of an oval form, and interspersed at its inferior part with furrows and incrustations, hard and resisting in some parts, and soft, but without fluctuation, in others, of a blackish brown colour. At the middle and fore part an oblong aperture, surrounded with a thick and callous border formed by the pressure, was observed. This aperture led to the urethra, which passed upwards and backwards towards the pubis. The corpora cavernosa were felt anteriorly at the neck of the tumour, and the testes on the sides, or rather towards the back part; these last seemed sound, and the spermatic chords were elongated and enlarged, and the arteries, whose pulsation was readily perceived, seemed to have enlarged their calibre; the skin of the abdomen was stretched to accommodate itself to the tumour, and the hair of the pubis was considerably below that region, so much, indeed, that the navel was on the pubis.

This enormous mass, which was supported by a suspensory, produced no other inconvenience, than to impede by its weight the motions of progression.

CASE

### CASE III.

An husbandman of Upper Egypt had a sarcocele for twelve or fifteen years, which was daily augmenting. At the time I saw him at Cairo, his tumour was enormous, and weighed near one hundred pounds; it descended nearly to his feet, separating the legs; it was of an oval form and of a brownish colour, unequal on its surface, and interspersed with incrustations; like the sarcocele of Ibrahim, the prepuce was in the middle of the anterior part, and the testicles on its sides, or towards the superior portion. After having been treated by the physicians of his country, he consulted an English physician who was travelling in Egypt; in hopes of a perfect cure, he consented to the application of an actual cautery; but the repeated use of this remedy gave him no relief, and the tumour continued in the same state. Some years after he consulted a Spanish physician, who also was journeying in Egypt, who plunged a knife deeply into the tumour, persuaded that it was hydrosarcocele, but there issued only a little blood, and the disease, so far from yielding to this operation, became worse and larger. These two operations, the patient said, were performed without his suffering much pain; and no accident or inconvenience was produced. The cicatrices were yet tender when I first saw him at Cairo, and he was disposed to submit to its extirpation, which I advised, but the same impediments, as in the former case, prevented my performing it.

### CASE IV.

Hamet Fatomi, thirty years of age, wife of a labourer of Cairo, came into the civic hospital on account of two tumours, which she had had for many years. These tumours seemed to have their origin in the external labia, for there was no vestige of these parts to be seen, nor of the nymphæ; they were nearly of the same size, were placed by the side of each other at the entrance of the vagina, and each of them resembled the head of an infant, rugous and unequal on the greatest part of their circumference, smooth on the inner part and of a violet colour; their prominent sides, or rather their base, was covered with pustulous incrustations, like the sarcocele of Ibrahim, and discharged a similar fœtid ichorous fluid, they were attached by small roots to the ischium and pubis, were hard, insensible, and like scirrhus, measured about thirteen inches each in circumference, four inches in diameter, and seven inches long.

The woman, who was of a sickly constitution, had an incipient cipient elephantiasis. Her lips were thick and of a lead colour, her gums pale and ulcerated, sorrowful appearance of the eyes and countenance, and disposed to melancholy; the digestive functions, however, went on well. I attributed this affection to the incipient elephantiasis, and it is remarkable that she had never menstruated regularly.

I proposed to extirpate the tumour, and began to give her remedies which I had already employed, with benefit, in elephantiasis. In six weeks her limbs and lips were less swelled, and nearly in their natural state; she had become stronger, the tumours were somewhat softer, the discharge which flowed from the ulcerated scabs had become less fœtid, in short, I considered her in a fit state for the operation.

The necessity of extirpation in this case, and in the case of Ibrahim, was acknowledged in a consultation, and the operation was fixed for the next day, when the order arrived for me to repair with the army to Alexandria, and obliged me to abandon both of these cases.

